# Herbal antioxidants in dialysis patients: a review of potential mechanisms and medical implications

**DOI:** 10.1080/0886022X.2021.1880939

**Published:** 2021-02-17

**Authors:** Masoumeh Asgharpour, Amirhesam Alirezaei

**Affiliations:** a Department of Nephrology, Rouhani Hospital, Babol University of Medical Sciences, Babol, Iran; b Department of Nephrology, Shahid Modarres Hospital, Shahid Beheshti University of Medical Sciences, Tehran, Iran

**Keywords:** Dialysis, oxidative stress, inflammation, herbal antioxidant, silymarin, curcumin, resveratrol, emodin, quercetin

## Abstract

The consumption of exogenous antioxidants isolated from herbal extracts has shown beneficial effects on ameliorating dialysis-related complications through debilitating oxidative stress and inflammatory process. Many clinical studies available in public databases have reported the improved consequences of dialysis in patients supplemented with herbal antioxidants. Exploration of such data offers great possibilities for gaining insights into the potential mechanisms and medical implications of herbal antioxidants. In this work, the mechanisms and implications of some famous bioactive substances including silymarin, curcumin, resveratrol, emodin, and quercetin on the consequences of dialysis in chronic kidney disease (CKD) patients were explored. The protective features of silymarin are due to the flavonoid complex silybin. Curcumin is an active element from the root of curcuma longa with extensive beneficial properties, including antioxidant, anti-inflammatory activity, and inhibitory effects on cell apoptosis. Resveratrol can reduce the oxidative stress by neutralization of free radicals. Emodin is known as a natural anthraquinone derivative isolated from Chinese herbs. Finally, quercetin has been reported to exhibit several properties including antioxidant, anti-diabetic, analgesic, antihistaminic, antiviral, cholesterol reducer, and renal hemodynamic modulator. However, potential mechanisms and medical implications of the aforementioned herbal antioxidants seem to be more complicated, that is, more studies are required in this field.

## Introduction

Chronic kidney disease (CKD), a public health problem, is featured by progressive renal dysfunction which lasts for more than three months [1,2]. Depending on the country, it may influence nearly 10% of the population and involves both males and females [3]. Patients with CKD are characterized by a gradual kidney function reduction or even lose over time due to progressive interstitial fibrosis [4]. In 2010, renal replacement therapy (RRT) was used for approximately 2.62 million patients worldwide which are expected to double by 2030 because of increasing the risk of obesity and diabetes mellitus among the different population and population aging in many countries [5]. The morbidity and mortality rate of dialysis patients remain high due to its complications such as cardiovascular disease [6,7].

Oxidative stress, a pathological state, is associated with overproduction of reactive oxygen species (ROS) and reactive nitrogen species (RNS), which reduce the scavenging capacity of antioxidant systems [8]. Enhanced oxidation of lipids, proteins, and DNA which has been shown in the CKD patients from early stages may result to organ damage [9]. The progression of CKD to ESRD is related to the enhanced oxidative stress due to decreased kidney function induced accumulation of uremic toxins which leads to exacerbation of ROS production and inflammatory responses and increases the risk of related complications [10]. Oxidative stress has also an important role in the promotion of atherosclerosis in ESRD patients [11]. Moreover, oxidative stress and inflammatory state result in peritoneum fibrosis in peritoneal dialysis (PD) patients [12].

A growing body of evidence suggests that oxidative stress is particularly prominent in patients undergoing dialysis [13]. Importantly, dialysis also increases ROS production and leads to oxidative stress and inflammation through different mechanisms [14].

It has been suggested that the amelioration of oxidative stress and inflammatory condition in dialysis patients seem to improve long-term survival in several kinds of research [15]. The antioxidant supplementation mainly from natural sources is an emerging strategy to attenuate oxidative stress and subsequent inflammation in pathologic conditions [16]. There is some evidence regarding the protective role of these agents in the management of CKD and dialysis induced oxidative stress and inflammation [17,18].

The present study aimed at evaluating the efficiency of antioxidant supplementation with natural sources in the management of different types of RTT mainly HD and PD induced oxidative stress, inflammation and, subsequent complication. Furthermore, this study investigated the underlying molecular mechanisms of these antioxidants.

## Underlying mechanisms for dialysis induced impairments in CKD patients

Traditional risk factors cannot explain the underlying mechanisms of CKD and its related cardiovascular diseases [19]. Both oxidative stress and chronic inflammatory state are important factors in the pathophysiology of dialysis-related complications such as, cardiovascular complications and also enhanced the mortality rate in these patients [11,20]. Oxidative stress, a novel nontraditional risk factor, and an important pathogenic mechanism is associated with an imbalance between the production of ROS and RNS and antioxidant systems to scavenge the free radicals [11,21]. It is a common cause of cellular structural alterations such as DNA damage and oxidation of lipids and proteins [22], which lead to apoptosis or cellular necrosis [23]. Oxidative stress also plays a major role in the progression of CKD to ESRD, particularly in dialysis patients [17,24].

The underlying mechanisms to explain the sources of enhanced oxidative stress and inflammation have not been revealed yet. Enhanced oxidative stress as well as increased mortality rate has been proven in both HD and PD patients [25,26]. Different mechanisms seem to induce enhanced ROS production and subsequent oxidative stress, as well as inflammation in dialysis patients such as, dialysis membrane induced activation of leukocytes, dialysis-induced removal of low molecular weight molecules of the antioxidant system, and restriction of fruit and vegetable consumption in these patients [14].

Several factors are considered in the oxidative stress-induced pathologic condition in HD and PD patients. Reports have indicated that there must be different treatment procedure in the oxidative stress status between HD and PD [25,27]. Oxidative stress in dialysis patients is more than that in CKD patients, similarly, Oxidative stress in HD patients is more than that in PD patients [12]. The type of dialysis membrane, intravenous administration of iron, heparin use, activation of platelets and leukocytes, and most importantly lack or decrease in urine volume, as well as some degree of malnutrition are responsible for the production of oxidation products in HD patients [17].

In ESRD patients, the enhanced ROS productions and lipid peroxidation of blood, reduced glutathione peroxidase (GPx), superoxide dismutase (SOD), and catalase (CAT) activities due to a membrane dialyzer during HD treatment were reported, which indicate the importance of membrane types especially biocompatible ones [28]. So, it seems that the HD procedure may be associated with antioxidant system impairment. The blood exposure to dialysate as well as dialyzer membranes within minutes following HD initiation seems to trigger activation of complement factors, platelets, polymorphonuclear (PMN) leukocytes, and consequently causes the overproduction of ROS [29]. Progressively increased PMN leukocytes stimulation has been reported as a prominent factor in HD patients during HD sessions [30]. Moreover, myeloperoxidase as an active enzyme in the generation of reactive species is stored within PMN leukocytes and is a marker of HD bio-incompatibility and oxidative stress which can be mobilized rapidly and extensively into circulating blood by exogenous heparin, indicating the myeloperoxidase-oxidative stress-heparin interaction in HD patients [31]. Also, increased lipid peroxidation products within 30 min of HD initiation have been hypothesized to be related to the activation of complement factor or heparin-induced production of free fatty acids [32]. HD patients were also reported to have significantly reduced plasma levels of antioxidants including vitamin (Vit) C, GSH-Px, and selenium, which implicates exacerbation of oxidative stress by the HD procedure [30].

Inflammation, a strong predictor of the poor outcome in dialysis patients [33], plays a major role in uremia-related morbidity and mortality in HD patients [34,35]. Inflammation in patients on dialysis may be associated with cellular responses to oxidative stress. Other factors are categorized as 1) tissue related factors e.g. hypoxia, fluid, and sodium overload; 2) gut dysbacteriosis and immune system dysfunction; 3) retention of uremic toxins, such as advanced glycation end-products (AGEs), indoxyl sulfate, and calcioprotein particles; and finally 4) dialysis membranes and central venous catheters as external factors [35]. HD procedure produced inflammation increases long-term morbidity and mortality in ESRD patients. The activation of interleukins and anaphylatoxins stimulates the nicotinamide adenine dinucleotide phosphate (NADPH) oxidase, an enzyme that plays a critical role in overproduction of ROS, and consequently leads to leukocyte activation, cytotoxicity, and subsequent organ failure [36]. Hyper-inflammation in chronic HD patients leads to cardiovascular disease and increases the mortality rate [37,38].

The accumulation of oxidative products in PD patients depends on PD solution characteristics such as, increased osmolality, low PH, elevated lactate levels, and products of glucose degradation [12,14]. Bio-incompatibility of conventional PD solutions has been revealed to form oxidation carbonyl products, leading to dramatic structural and functional changes in the peritoneal membrane due to protein oxidation and AGEs accumulation [39]. Similarly, PD treatment has been reported to increase the peritoneal nitric oxide synthase (NOS) activity in biopsies which is responsible for the formation of the prooxidant NO. In long-term PD patients, NOS overexpression and NO overproduction may underlie the pathological alterations of the peritoneal membrane including enhanced vascular endothelial growth factor activation, AGEs accumulation, and subsequent peritoneal membrane calcification [40].

On the other hand, the chronic inflammatory responses induced by the long-term PD use can be associated with structural and functional changes in the peritoneal membrane and exacerbation of the oxidative products [41]. In early phases of PD treatment, even within the first hour after the initiation, elevated levels of serum inflammation factors such as interleukin-6 (IL-6), was reported due to the bio-incompatibility of the conventional PD solution [42,43].

## Natural antioxidants

In the human body, the antioxidant system can scavenge free radicals and keep the balance between oxidative products and anti-oxidative activities [44]. Antioxidants are classified into two types of endogenous which are natural defense mechanisms produced by the human body; and exogenous which can be found in supplements and food [45]. Endogenous antioxidants can be further categorized as enzymatic or non-enzymatic. Antioxidants also are classified as water- or fat-soluble molecules [46]. Endogenous antioxidant enzymes such as, SOD, CAT, and GPx can suppress the toxic ROS production and regulate the body homeostasis [47].

Medicinal plants play a very important role in the protection of human health not only during ancient age but also in modern culture. The majority of exogenous antioxidants are isolated from medicinal plants and foods including vegetables, fruits, cereals, nuts, flowers, legumes, beverages, fungi, spices, etc. [48]. Generally, the natural antioxidants from plant materials are primarily categorized as follows: (1) polyphenols, including flavonoids, phenolic acids, lignans, anthocyanins, and stilbenes; (2) carotenoids, including xanthophylls and carotenes, and (3) vitamins, particularly vitamins E and C [49]. A variety of important biological activates such as anti-inflammatory, antiviral, antibacterial, anti-aging, and anticancer activates have been revealed for these natural antioxidants, particularly for polyphenols and carotenoids [50]. The administration of exogenous antioxidants may attenuate the damage induced by oxidative stress in organs through suppression of the initiation or progression of oxidative chain reaction, thus, they can act as free radical neutralizer agents [51].

The beneficial effects of medical plants extract on the renal damages were confirmed via anti-inflammatory, anti-apoptotic, and anti-oxidative properties [52–54]. Moreover, herbal medicine was successfully used to ameliorate the consequences of patients on dialysis [55,56]. However, the administration of plant-derived natural products has high efficiency compared to medical plants, suggesting these bioactive components as a source of plant-derived drugs with lower side effects [57]. Additionally, naturally occurring substances are different from chemical drugs in various aspects including their cost, administration, production, and clinical efficacy [58]. In this review, we tried to investigate the effects of some famous bioactive substances on the consequences of dialysis in CKD patients.

## Silymarin

Silymarin is a flavonoid isolated for the first time in 1968 from the seed extract of milk thistle plant [59] which is mainly a mixture of lignin-derived flavonols, including silybin, silydianin, silychristin, and isosilybin [60]. Silymarin has a relatively safe profile without any side effects [61] which can neutralize harmful free radicals and inhibit oxidation of lipid cell membranes [62]. The protective features of silymarin are due to the flavonoid complex silybin, which is a potential antioxidant agent and scavenge ROS produced in normal metabolic processes and during the neutralization of toxic substances. Silymarin also enhances concentrations of endogenous antioxidants such as, GSH-Px, and SOD [63]. Because of anti-fibrotic and anti-inflammatory properties, silymarin is considered as a natural agent for the prevention and treatment of liver and kidney disorders [64,65].

The nephron-protective effect of silymarin has been confirmed in several studies. Based on the experimental results, silymarin may prevent cisplatin and adriamycin-induced renal injury via its antioxidant and anti-inflammatory properties [66,67]. Also, other animal studies reported that silymarin was able to lower functional and structural kidney cell alterations induced by ischemia/reperfusion injury [68,69]. However, in an *in vitro* study, silymarin could not decrease lipid and DNA oxidation caused by glycerol in rat renal cells [70]. To show the nephroprotective features in human, 60 patients with diabetic kidney disease received the placebo or silymarin (420 mg/daily) in a randomized, controlled, double-blind study. The results showed a significant reduction in urinary TNF-a and MDA levels and also albuminuria was improved in silymarin group, emphasizing the anti-inflammatory and anti-oxidative properties of this agent [71].

Due to the beneficial effects of silymarin on the kidney, this antioxidant also was used to prevent the PD and HD induced complications. In a study to evaluate the efficiency of silymarin on HD patients, 80 HD patients were randomly assigned into four groups and received silymarin (420 mg/day), vit E (400 IU/day), silymarin + vit E or placebo for 3 weeks. The best results were reported in silymarin + vit E in which the serum levels of MDA were decreased and RBC levels of the GPx and hemoglobin levels were increased [72]. Furthermore, treatment of 15 chronic PD patients with silymarin (210 mg/day) for 8 weeks demonstrated significantly enhanced levels of hemoglobin as well as decreased levels of serum TNF-a, indicating the efficacy of silymarin in management of oxidative stress and inflammation in PD patients [73]. In a randomized clinical study, 50 ESRD patients undergoing PD were treated with silymarin (140 mg every 8 h) or placebo for two months and then, the biomarkers of oxidative stress in plasma and erythrocytes were measured. The activity of the antioxidant endogenous enzyme CAT in red blood cells and anemia due to hemoglobin values were improved in the silymarin group compared to placebo [74].

## Curcumin

Curcumin (C21H20O6), a hydrophobic polyphenol, is an active element from the root of curcuma longa with extensive beneficial properties, including antioxidant, anti-inflammatory activity, and inhibitory effects on cell apoptosis [75]. The safety and efficiency of this agent highlight its influence on the prevention and treatment of different types of human illnesses. As a free radicals scavenger, curcumin can directly neutralize the ROS by donating its hydrogen ions and indirectly overexpress the antioxidant system enzymes such as, CAT, SOD, and GSH [76]. Additionally, this antioxidant applies its efficiency by inhibiting the nuclear factor-kappa B (NF-kB) cell signaling pathway [77]. *In vitro* studies showed that preincubation of activated macrophages of rat peritoneum with curcumin inhibited the accumulation of ROS by macrophages [78] and might be a stronger inhibitor of lipid peroxidation [79].

The beneficial effects of curcumin on animal models of nephropathy have been confirmed in several studies. The supplementation of curcumin (75 mg/kg/day) has been reported to lower inflammation, oxidative stress, and renal fibrosis in the remnant kidney via the nuclear factor-erythroid-2-related factor 2 (Nrf2)-keap1 pathway [80]. Furthermore, curcumin (daily by gavage at doses of 37.5, 75, and 150 mg/kg for 35 consecutive days) could lower the CKD-like renal damage via inhibition of inflammatory cytokines (IL-1β, IL-6, and TNF-α), oxidative stress, fibrosis, and apoptosis [81]. Using similar mechanisms, the nephroprotective effects of curcumin have been revealed in animal models with nephrotoxicity and CKD [82–84]. On the other hand, a diet containing curcumin for eight weeks attenuated the release of lysosomal enzymes and eicosanoids in rat peritoneal macrophages [85].

Clinical experiments have been done to investigate the efficiency of curcumin on patients with kidney impairment. In an evaluation of curcumin supplementation (66 mg of curcumin per day) for 2 months in patients with diabetic CKD, 39% reduction in proteinuria and decreased serum levels of transforming growth factor-beta (TGF-β) and TNF-α were reported [86]. Similarly, curcumin supplementation decreased significant proteinuria in patients with lupus nephritis [87].

In limited studies, the antioxidant and anti-inflammatory effects of curcumin administration in patients undergoing dialysis are quite promising. In a clinical study, the anti-inflammatory effects of curcumin on HD patients using one capsule containing 500 mg turmeric with 22.1 mg active ingredient curcumin (3 caps/day for 12 weeks) was investigated. The results showed that curcumin with no adverse effects was able to decrease the plasma level of highly-sensitive C- reactive protein (hs-CRP), TNF-a, and IL-6, and to increase albumin levels in HD patients [88]. In a cohort study on 43 HD-dependent cadaver kidney recipients, curcumin supplementation for one month could lower the acute rejection rate and neurotoxicity within six months [89]. Another randomized, double-blind study on stable HD patients showed that treatment with curcumin (1500 mg/day) for two months resulted in significant enhancement of the CAT activity as well as erythrocytes concentration and reduced serum levels of MDA compared to the placebo group [90]. The results of a pilot randomized, double-blind, controlled study demonstrated that curcumin supplementation (100 mL of orange juice with 12 g of carrot and 2.5 g of turmeric after each dialysis session/week) for 3 months ameliorated the plasma levels of hs-CRP and the expression of Nrf2, NF-kB, NLRP3, and IL-1β in peripheral blood mononuclear cells in HD patients [91].

## Resveratrol

Resveratrol (3,5,4-trihydroxystilbene) is a plant polyphenolic compound found in red wine, grapes, berries, apples, blueberries, plums, peanuts, and other oil seeds [92]. Resveratrol can, directly and indirectly, reduce the oxidative stress by neutralization of free radicals and up-regulation of the endogenous antioxidant enzymes like, SOD, CAT, and GPx [93]. Resveratrol administration with anti-inflammatory and antioxidant features attenuated oxidative stress-mediated renal damage in animal models of septic nephropathy [94], ischemia/reperfusion acute kidney injury [95], nephrotoxicity induced by gentamycin [96], cisplatin [97], and cyclosporin [98], as well as diabetic nephropathy [99]. Different intracellular signaling molecules in kidney cells are affected by the administration of resveratrol including reduced oxidative stress, decreased levels of ROS and MDA, increased antioxidant enzyme activity, and improved mitochondrial biogenesis [100–103].

Furthermore, resveratrol has been reported to show inhibitory effects on IL-6 expression by stimulated peritoneal macrophages in mice model [104].

The clinical studies on resveratrol effects are limited. In a randomized, prospective, double-blind study, a twelve-week treatment with high-dose trans-resveratrol (450 mg/d) resulted in health benefits and improvement of ultrafiltration in PD patients via down-regulation of angiogenesis factors such as, vascular endothelial growth factor, angiopoietin-2, and fetal liver kinase-1 in peritoneal effluent induced by conventional lactate-buffered PD solutions, suggesting that resveratrol with high doses and longer duration of administration may provide beneficial effects in PD patients via angiogenesis-ameliorating impacts [105]. Conversely, the four-week administration of 500 mg/day was reported to have no significant effects [106]. In a randomized, double-blind, placebo-controlled trial, a total of 40 CKD patients with iron overload undergoing HD, were randomly assigned into the groups of (1) supplementation with resveratrol (500 mg) and Curcumin (500 mg); and (2) placebo. The supplementation group revealed the improvement in bone and muscle mass as well as circulating ferritin levels, confirming that this novel combination had beneficial effects in HD patients [107].

## Emodin

Emodin (3-methyl-1,6,8 trihydroxyanthraquinone) is known as a natural anthraquinone derivative isolated from Chinese herbs such as the root of rheum palmatum L [108]. In recent studies, a wide spectrum of pharmacological effects of emodin has been confirmed including the anti-inflammatory, anti-tumoral, antiviral, antibacterial, anti-allergic, chemo-preventive, anti-diabetic, and immunosuppressive activities [109]. Besides, anti-inflammatory and anti-proliferative features of emodin are related to its inhibitory effects on tyrosine kinase activity [110,111].

In several studies, the nephroprotective effects of emodin have been reported. Administration of emodin could successfully attenuate TGF-β1 and fibronectin synthesis in mesangial cells under diabetic condition via inhibition of the NF-κB signaling pathway [112], and also reduced renal hypertrophy and hyperfiltration in rat models of diabetic nephropathy [113]. Nephroprotective effects of emodin against diabetic renal injury in rat models were reported to be attributed to activation of PI3K/Akt/glycogen synthase kinase 3β signaling pathway suppression of inflammation, Bax/Caspase3 pathway [114], and phosphorylation of P38 MAPK [115], which mitigated podocytes apoptosis induced by endoplasmic reticulum stress via the inhibition of the PERK pathway [116]. As an inhibitor of the fibrotic process in CKD, emodin was revealed to suppress extracellular matrix formation via modulation of P38 and ERK1/2 pathways in TGF-β1-stimulated NRK-49F cells [117]. Additionally, emodin was reported to reduce the interstitial fibrosis via up-regulation of TIMP1 and Smad7 expression and down-regulation of MMP9, TGF-β1, and smurf in the kidney of rats [118,119].

Since the inflammatory responses and fibrosis mediated by peritoneal mesothelial cells which were exposed to the high dialysate glucose concentration during PD, emodin was reported to ameliorate the morphologic changes and chronic fibronectin synthesis in human peritoneal mesothelial cells induced by 30 mmol D-glucose [120]. Emodin also was shown to reduce glucose-induced matrix synthesis in human peritoneal mesothelial cells via inhibition of protein kinase C activation and cAMP response element-binding protein (CREB) phosphorylation, which confirms that emodin might be a potential target to prevent and treatment of glucose-induced pathological changes in the peritoneal membrane [121]. In another study, emodin ameliorates glucose-induced structural and functional alteration in human peritoneal mesothelial cells via inhibition of TGF β1 and fibronectin synthesis [120]. Emodin was proved to reduce the peritoneal fibrosis via inhibition of mRNA levels of Notch1, Jagged-1, and Hes-1 in peritoneal tissue in the rat model of PD [122].

## Quercetin

**Figure 1.**
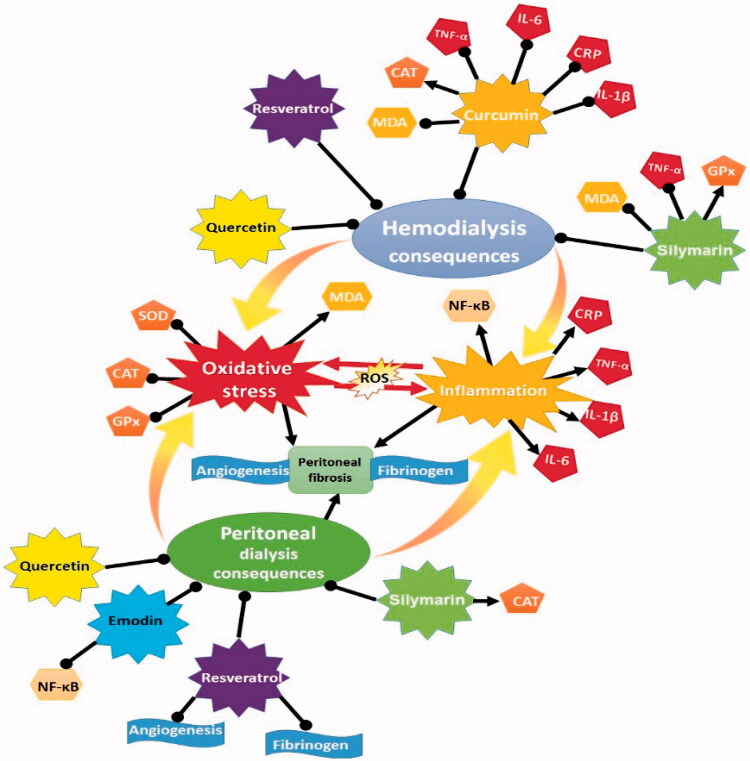
The underlying mechanisms of natural antioxidants against HD and PD consequences.

Quercetin (3, 5, 7, 3′ & 4′-pentahydroxy flavonol), an abundant vegetal flavonoid in the mediterranean diet, has been reported to exhibit several properties including antioxidant, anti-diabetic, analgesic, antihistaminic, antiviral, anti-inflammatory, cholesterol reducer, and renal hemodynamic modulator [123,124]. In an animal model of CKD, treatment with quercetin improved renal function and histopathological alterations through reduction of oxidative stress factors, serum levels of fibroblast growth factor 23, parathyroid hormone, inorganic phosphate as indicators of CKD development, and kidney inflammation [123]. Another study on rats showed that quercetin ameliorated methotrexate [125] and cisplatin-induced [126] renal damage by reducing apoptosis and oxidative stress. Chronic administration of quercetin also has supportive effects on cadmium-induced renal damage due to the antioxidant, anti-inflammatory, and vasodilator properties [127]. The nephroprotective effects of this flavonoid in an ischemia/reperfusion model also have been proven [128]. Also, in diabetic nephropathy, quercetin improves the renal function by suppression of progression of renal fibrosis and mammalian target of rapamycin (mTOR/p70S6) kinase (p70S6K) signaling which mediates renal tubular epithelial-mesenchymal transition [129]. In *in vitro* studies, the reno-protective effect of quercetin has been reported for tubular epithelial cells via removing the free radical and reducing the lipid peroxidation [130,131]. In addition, this agent has been shown to attenuate the TGFβ induced fibrosis in renal tubular epithelial cells via upregulation of PTEN and TIMP3, and suppression of miR21, which highlights the anti-fibrotic feature of quercetin for CKD patients [132]. In an *in vitro* study, quercetin has been shown to protect human mesothelial cells from structural changes against exposure to PD fluid through the reduction of lactate dehydrogenase [133] (Figure 1).

## Conclusion

Many clinical studies available in public databases have reported the improved consequences of dialysis in patients supplemented with herbal antioxidants. It means that, the consumption of exogenous antioxidants isolated from herbal extracts has shown beneficial effects on ameliorating dialysis-related complications through debilitating oxidative stress and inflammatory process. The mechanisms and implications of some famous bioactive substances including silymarin, curcumin, resveratrol, emodin, and quercetin on the consequences of dialysis in chronic kidney disease (CKD) patients were explored in this study. It seems that the beneficial effects of these bioactive substances are mainly related to their free radical savaging and anti-inflammatory activities. In other words, HD and PD processes are accompanied by complications that are related to different factors mainly oxidative stress and inflammation. The dialysate composition in PD and dialysis membrane in HD are associated with pathological alterations. Since several therapeutic approaches were developed to manage the PD and HD consequences, supplementation of dialysis patients with naturally occurring antioxidants seems to be fruitful. However, larger cohort studies with hard end-points and longer treatment periods are required to evaluate the effect of natural antioxidants on PD and HD outcomes.

## Abbreviations

ROS: reactive oxygen species
MDA: malondialdehyde
GPx: glutathione peroxidase
SOD: superoxide dismutase
CAT: catalase
TGF: transforming growth factor
IL: interleukin
NF-kB: nuclear factor kappa B
CRP: C- reactive protein
